# Impact of Supplemental Oxygen on Obstructive Sleep Apnea of Infants

**DOI:** 10.3390/children5030034

**Published:** 2018-03-02

**Authors:** Piyush Das, Rahul Kashyap, Suresh Kotagal

**Affiliations:** 1 Department of Psychiatry, Mayo Clinic College of Medicine, Rochester, MN 55905, USA; drpiyushdas@yahoo.com; 2 Saint Cloud Hospital Sleep Center, CentraCare Health, Saint Cloud, MN 55905, USA; 3 Department of Anesthesiology & Perioperative Medicine, Mayo Clinic College of Medicine, Rochester, MN 55905, USA; kashyap.rahul@mayo.edu; 4 Multidisciplinary Epidemiological and Translational Research in Intensive Care (M.E.T.R.I.C.), Mayo Clinic, Rochester, MN 55905, USA; 5 Center for Sleep Medicine, Mayo Clinic College of Medicine, Rochester, MN 55905, USA; 6 Division of Pediatric Neurology and Center for Sleep Medicine, Mayo Clinic College of Medicine, 200 First Street SW, Rochester, MN 55905, USA

**Keywords:** infants, sleep apnea, obstructive, oxygen

## Abstract

Treatment options may be limited for infants with obstructive sleep apnea when there is no surgically correctable upper airway lesion. We therefore evaluated, retrospectively, the efficacy of low-flow oxygen as a therapeutic option for infant obstructive sleep apnea. We reviewed the medical charts of 23 infants who had undergone a therapeutic trial of low-flow oxygen during polysomnography. Split-night polysomnography was used in 21/23 subjects while 2/23 had undergone two separate, full-night polysomnography sleep architecture and respiratory findings on the baseline polysomnogram segment that was obtained in room air were compared with the segment on low-flow oxygen (0.25–1 L/min). Wilcoxon signed rank or McNemar’s test were used as indicated for comparing apnea hypopnea index and measures of sleep architecture at baseline and with oxygen therapy. The mean (±SD) age of subjects was 4.8 (±2.7) months, with 52% being males. The median apnea hypopnea index fell from a baseline of 18 (range 7–43) to 3 (range 1–19; *p* = 0.001) on oxygen. The baseline median obstructive/mixed apnea index decreased from 2 (range 1–16) to 1 during oxygen therapy (range 0–1; *p* = 0.003). Additionally, a significant decrease in central apnea index (median interquartile range (IQR) 1 (0–2) vs. 0 (0–1), *p* = 0.002) was noted. Sleep efficiency remained unaffected, while O_2_ saturation (SaO_2_) average and SaO_2_ nadir improved on oxygen. We were able to confirm the utility of low-flow oxygen in reducing central, obstructive, and mixed apneas and improving average oxygen saturation in infants with obstructive sleep apnea (OSA).

## 1. Introduction

Obstructive sleep apnea (OSA) seen during infancy is often related to gastro-esophageal reflux, laryngotracheomalacia, craniofacial anomalies, or neuromuscular disorders [1,2]. Significant adenotonsillar hypertrophy, a common etiology in older children, is encountered less often, hence adenotonsillectomy is generally a less frequent treatment option. Even when adenotonsillectomy is performed for relief of obstructive sleep apnea in infancy, it seems to benefit more those patients who lack comorbidities [3]. Positive airway pressure (PAP) is utilized in older children and adults, but these devices are not approved by the Food and Drug Administration for use in those weighing less than 13 kg. Oxygen therapy is used empirically to treat OSA in this age group. Supplemental oxygen use has been reported for improving cardiorespiratory stability in asymptomatic preterm infants [4]. Given the paucity of evidence supporting the efficacy of oxygen in treating infant OSA, we report on our experience in this regard, with an emphasis on changes in sleep-related respiratory variables.

## 2. Materials and Methods

A retrospective medical chart review was conducted at a tertiary referral sleep center that was accredited by the American Academy of Sleep Medicine. The study protocol was approved by the Institutional Review Board (IRB) of Mayo Clinic, Rochester, MN, USA on 1 July 2011 (No. 10-007218). The medical charts were reviewed of all infants who had undergone a therapeutic trial of low-flow supplemental oxygen during attended nocturnal polysomnography in our sleep laboratory. The subjects had been initially referred to our pediatric otolaryngology service for assessment of potential surgically correctable lesions underlying OSA. The study period was 2004 to 2010. The diagnosis of OSA was made on the basis of presence of one or more obstructive apneas/hypopneas per hour of sleep on room air in infants [5]. Consecutive infants who also had been evaluated by the otolaryngology service and were deemed to not be candidates for surgical treatment of OSA (determined not to have an anatomic, surgically correctable upper airway lesion) were included. Infants with congenital heart disease were excluded. They were provided low-flow supplemental oxygen trial during nocturnal polysomnography if there was significant recurrent apnea (obstructive, mixed, or central) and oxygen desaturation. Underlying medical diagnoses on these patients are listed in Table 1. These infants underwent either two successive nocturnal polysomnograms (one night on room air and the second night on supplemental oxygen) or a single night polysomnogram that was split into two halves—baseline segment without supplemental oxygen, and a low-flow supplemental oxygen therapeutic trial segment. A criterion for split night study was the capture of both non-rapid eye moment (NREM) and rapid eye moment (REM) sleep in the baseline, diagnostic segment. The diagnostic segment (without oxygen) was always the first segment. The oxygen therapy segment was always in the second half of the night. Since REM sleep is generally more abundant in the second half of the night and since OSA is frequently worse in REM than in NREM sleep, we anticipated the beneficial effect of oxygen therapy is this half. Supplemental low-flow oxygen consisted of 0.25 to 1 L/min. It was provided during attended polysomnography using a nasal cannula. 

Primary outcomes of interest were number of apneas (obstructive/mixed) per minute, combined apnea/hypopnea index, and sleep efficiency. Secondary outcomes measured were number of central apneas, total sleep time, and average O_2_ saturation (SaO_2_) and SaO_2_ nadir during sleep, with and without supplemental low-flow oxygen.

Data points of interests were extracted using a standardized data extraction form. Information about the following variables was collected: age, gender, total sleep time, obstructive/mixed apneas per minute, apnea-hypopnea index, hypopnea index, central apneas/minute, sleep efficiency, average SaO_2_ and SaO_2_ nadir, periodic leg movement (PLM) index, and PLM arousal index. 

Continuous variables are expressed as mean ± standard deviation (SD) or median with interquartile range (IQR) based on the distribution. Categorical variables are expressed using frequency and % frequency. Wilcoxon signed rank or McNemar’s tests were used as appropriate, to compare sleep-related parameters with and without low-flow oxygen supplementation. Statistical analysis was done using JMP 10.0 statistical package (SAS Inc., Cary, NC, USA).

## 3. Results

During the period of 2004–2010, a total of 23 infants underwent trial of low-flow oxygen supplementation during polysomnography—21 underwent a split-night study and two had polysomnograms on two separate nights within a one-month period. The mean (±SD) age of the subjects was 4.8 (±2.7) months; 52.2% were male and 17 (74%) were Caucasian. A total of 10 (44%) infants were born prematurely (<37 weeks gestation), with mean (±SD) age of 32 (±4.4) weeks. A total of 8 (35%) infants had gastroesophageal reflux and 13 (56%) had craniofacial anomalies. None of these had neuromuscular disorders. The awake SaO_2_ median (IQR value) was 96% (92–99%) and the median duration of low-flow oxygen supplementation (IQR) was 203 (136–334) min. 

Table 2 shows the comparison of important polysomnogram (PSG) parameters in room air versus on low-flow supplemental oxygen. In summary, low-flow oxygen use led to a significant decrease in obstructive/mixed apneas (*p* = 0.003), hypopnea index (*p* < 0.001), and combined apnea-hypopnea index (*p* < 0.001). A subset analysis found that the decrease in obstructive/mixed apneas with oxygen was statistically significant for both REM and NREM sleep. A decrease in central apneas index was also noted (*p* = 0.002). The average percentage of SaO_2_ improved with low-flow oxygen supplementation (*p* = 0.003), as did the SaO_2_ nadir (*p* < 0.001). The overall arousal index remained unchanged (15 vs. 19, *p* = 0.8), but the proportion of breathing-related arousals decreased significantly with supplemental O_2_ (36% vs. 15%, *p* = 0.005). There was no significant change in sleep efficiency (*p* = 0.34). An increase in PLM index (*p* = 0.001), and PLM arousal index (*p* = 0.002) on low-flow supplemental oxygen was statistically significant. 

Out of 23 infants, 10 (44%) had been born prematurely (<37 weeks gestation). A subset analysis showed that the combined apnea/hypopnea index and hypopnea index improved significantly with O_2_ supplementation in these prematurely born infants. There was no worsening of the central apnea index in this premature infant subgroup. For the obstructive/mixed apnea index, there was a non-significant trend towards improvement in NREM and REM sleep, whereas TST was significantly improved (Table 3).

## 4. Discussion

The findings of the current study suggest that use of low-flow supplemental oxygen in infants with OSA may decrease obstructive and mixed apneas and hypopneas. We also observed a decreased number of central apneas on oxygen treatment. Use of low-flow supplemental oxygen also increased the average SaO_2_ and SaO_2_ nadir during sleep. There was no change in sleep efficiency. In patients who underwent two night sleep studies (one on room air and second on supplemental oxygen), comparisons were thought to be valid, since there is evidence that apnea index and duration remains consistent from one night to another [6,7] and both of these study nights were within one month time frame.

Also, since infants spend a high proportion of their total sleep time in REM sleep, obstructive sleep apneic/hypopnea events can be problematic given the tendency for hypotonic collapse of the upper airway and impaired central responses to carbon dioxide accumulation during this state of sleep [8,9]. It is possible that hypoxia resulting from the sleep apnea episodes could further increase the tendency for hypotonic collapse of the upper airway dilator muscles and exacerbate the OSA [10]. Aljadeff et al [11] found that supplemental oxygen administration improved oxygen measurements, and reduced hypopnea density, hypopnea index and paradoxical breathing in children aged 1–16 years with moderate to severe OSA. An editorial by Brouillette et al. [12] which critiqued Aljadeff study mentions being careful with supplemental O_2_ administration in children with OSA related severe sequelae like congestive heart failure and cor pulmonale, sleep-related hypoventilation and disorders of central nervous system.

In a randomized double blind study, Marcus et al. [13] showed that supplemental O_2_ during sleep in children with OSA resulted in improved oxygenation without impacting the duration of apneas. However, they did recommend monitoring the end-tidal carbon dioxide (EtCO_2_) levels in children with OSA receiving O_2_ therapy, since two of their 23 subjects did show worsening of EtCO_2_. In their study, the limitations included exclusion of infants as study subjects and inclusion of OSA patients secondary to adenotonsillar hypertrophy only, which prevents extrapolation of results of this study to children with other diseases, especially children with neurologic disease with abnormal central ventilatory control. In a retrospective study by Kirk et al. [14] looking at various treatment modalities for sleep-disordered breathing in children (including infants) and young adults with myelomeningocele, supplemental O_2_ was not associated with any benefit in two cases of OSA but improved central apnea and central hypoventilation in other cases. The authors did not specify the age of patients in whom the supplemental O_2_ was used. They did caution about worsening of hypercarbia with O_2_ supplementation. 

Simakajornboon et al. [4] utilized supplemental oxygen to stabilize cardio-respiratory function in preterm infants. In their prospective study of 23 premature infants, frequent respiratory and cardiac events were noted in the laboratory setting. Supplemental oxygen decreased the amount of apneas (3.8 ± 2.4 events/h on supplemental O_2_ vs. 11.1 ± 6.4 events/h on room air), the duration of time spent in periodic breathing (1.8 ± 2.9% on supplemental O_2_ vs. 6.7 ± 8.9% on room air), and bradycardia events, while improving overall oxygen saturation. Supplemental oxygen use in their study did not worsen ventilation. The investigators felt that that improved oxygenation facilitated respiratory control despite reduction in the hypoxic drive induced by oxygen supplementation. Additionally, they observed an increase in slow-wave sleep and a reciprocal decrease in active sleep, which led them to propose that supplemental oxygen may facilitate growth via the augmentation of slow-wave sleep and consequent increase in growth hormone pulsatile release. 

Finally, a recent study [15] concluded that CPAP-intolerant school-aged children with OSA have good outcomes with high-flow, heated, and humidified air.

Our findings suggest a potential role for low-flow supplemental oxygen as a non-invasive and effective therapeutic alternative for OSA in infants. Increasing average SaO_2_ during sleep and blunting SaO_2_ nadir can prevent hypoxia-related serious cardio-respiratory adverse effects such pulmonary hypertension and cor pulmonale. This measure can also serve as a palliative strategy to prevent obstructive events and its complications or as a bridging therapy until a more definitive treatment like surgery can be performed to relieve the cause of OSA [16].

Various mechanisms have been suggested for the decrease in apnea/hypopnea density and apnea/hypopnea index with the use of supplemental oxygen. They include a decrease in the contracting force of the diaphragm with correction of hypoxemia-induced increased respiratory drive, as this contracting force predisposes to upper airway collapse [8]. There may also be improvement in geniohyoid muscle tone with correction of long-standing hypoxemia [11,17,18]. Supplemental oxygen via nasal cannula has also been hypothesized to provide a small amount of positive airway pressure effect and decrease in ventilatory muscle fatigue [11,19,20]. Additionally, supplemental O_2_ has been shown to improve apnea/hypopnea index (AHI) in OSA adults with high loop gain (unstable ventilatory control system due to increased chemo responsiveness) by reducing the loop gain by virtue of its ventilatory stabilizing properties via reduction in peripheral chemo-responsiveness [21]. 

There are some limitations of this study—it has the usual disadvantages of a retrospective study, like limited data availability and difficulties in controlling bias and confounders. Additionally, due to the retrospective nature of this study, the sequence of the intervention (i.e., O_2_ supplementation) could not be randomized, and most sleep studies were done as a split study, which precluded accurate assessment of sleep architecture and cardiorespiratory parameters. One of the potential adverse effects of supplemental O_2_ is alveolar hypoventilation, as measured by EtCO_2_. In our study, the EtCO_2_ data was not consistently available, due to which the safety of supplemental O_2_ could not be entirely ascertained. Since the study population was mainly Caucasian, the results may not be generalizable to other ethnic groups. Further, improved breathing-related parameters in the sleep laboratory do not necessarily imply that function in the home setting will also improve.

## 5. Conclusions

In a sleep laboratory population, low-flow supplemental oxygen was an effective treatment for obstructive sleep apnea during infancy. Possible mechanisms include alleviation of hypoxemia-induced hypotonia of the upper airway dilator muscles, positive airway pressure effects, decreased ventilatory muscle fatigue, decrease in contracting force of the diaphragm, and decrease in loop gain. Further prospective multicenter trials are warranted to reinforce the beneficial role of low-flow supplemental oxygen in the treatment of OSA as well as in central sleep apnea.

## Figures and Tables

**Table 1:** Patient demographics and underlying medical diagnoses.

Patient No.	Gender (Male = 0, Female = 1)	Age at Polysomnography (Months)	Underlying Medical Diagnosis
1	1	6	Trisomy 2 Mosaicism, Gastro-Esophageal Reflux
2	0	2	Trisomy 21, Cleft Palate, Laryngomalacia
3	1	3	Trisomy 21, Gastro-Esophageal Reflux
4	0	7	Gastro-Esophageal Reflux, Laryngomalacia
5	1	3	Gastro-Esophageal Reflux, Cranio-Facial Anomaly
6	0	7	Achondroplasia
7	0	2	Prematurity, Apparent Life Threating Episodes (ALTE)
8	0	3	Prader–Willi Syndrome
9	1	0.25	Prader–Willi Syndrome
10	0	7	Gastro-Esophageal Reflux, Tracheomalacia
11	1	5	Laryngomalacia
12	0	2	Gastro-Esophageal Reflux
13	0	7	Cranio-Facial Anomaly, Laryngomalacia
14	0	4	Cornelia De Lange Syndrome
15	1	5	Trisomy 21
16	1	10	Prematurity, Bilateral Cerebral Infarct, Gastro-Esophageal Reflux
17	0	6	Laryngomalacia
18	1	4	Neural Tube Defects, Chiari type II Malformation
19	0	7	Prematurity, Tracheomalacia, Bronchopulmonary Dysplasia
20	0	3	Chromosomal 4q Microdeletion, Central Sleep Apnea
21	1	11	Prematurity, Central Sleep Apnea
22	1	2	Microcephaly, Gastro-Esophageal Reflux
23	1	4	Pierre Robin Sequence

**Table 2:** Comparison of sleep-related parameters during polysomnography in room air vs. in low-flow supplemental oxygen.

Variable Median (IQR) (*n* = 23)	Without Oxygen	With Oxygen	*p*-Values
SaO_2_ Average (%) during Sleep	96 (92–98)	98 (94–99)	0.003 *
SaO_2_ Nadir (%)	81 (75–89)	91 (82–95)	<0.001 *
Central Apneas in:			
NREM	0 (0–2)	0 (0)	0.039 *
REM	0 (0–3)	0 (0)	0.006 *
TST	1 (0–2)	0 (0–1)	0.002 *
Obstructive/Mixed Apneas in:			
NREM	2 (0–8)	1 (0–1)	0.001 *
REM	3 (0–15)	0 (0–4)	0.036 *
TST	2 (1–16)	1 (0–1)	0.003 *
Combined AHI in:			
NREM	12 (5–33)	2 (1–13)	<0.001 *
REM	36 (12–64)	7 (2–35)	<0.001 *
TST	18 (7–43)	3 (1–19)	<0.001 *
Hypopnea Index in:			
NREM	7 (1–19)	1 (0–9)	0.003 *
REM	19 (7–44)	3 (0–13)	0.001 *
TST	12 (4–31)	1 (0–11)	<0.001 *
Arousal Index (*n* = 22) % Breathing-Related	15 (12–26) 36 (15–58)	19 (15–26) 15 (4–42)	0.8 0.005 *
REM Sleep Proportion	0.24 (0.13–0.28)	0.22 (0.15–0.28)	0.76
PLM Index,	6.8 (0–25.8)	23.4 (0–50.2)	0.001 *
Abnormal PLM Index, *n* (%)	14 (61)	11 (48)	0.26
PLM Arousal Index	1.3 (0–4.5)	6.2 (0–12.4)	0.002 *
Sleep Efficiency (%)	88 (80–91)	80 (76–94)	0.34

IQR: Interquartile Range; SD: Standard deviation; SaO_2_: Arterial oxygen saturation; NREM: Non-rapid eye moment; REM: Rapid eye movement; TST: Total sleep time; AHI: Apnea-hypopnea index; PLM: Periodic leg movement. * Statistically significant.

**Table 3:** Comparison of sleep apnea and hypopnea indices during polysomnography in room air versus in low-flow supplemental oxygen in children born preterm.

Variable Median (IQR) (*n* = 10)	Without Oxygen	With Oxygen	*p*-Values
Central Apneas in:			
NREM	1 (0–2)	0 (0)	0.06
REM	0 (0–6)	0 (0–1)	0.12
TST	2 (0–4)	0 (0–1)	<0.05 *
Obstructive/Mixed Apneas in:			
NREM	2.5 (0–12)	1 (0–4)	0.07
REM	5.5 (0–28)	0.5 (0–12)	0.1
TST	5.5 (1–21)	1 (0–6)	<0.05 *
Combined AHI in:			
NREM	25 (6–35)	2 (1–14)	<0.01 *
REM	45.5 (23–77)	7 (5–35)	<0.05 *
TST	32 (14–46)	3 (2–20)	<0.01 *
Hypopnea Index in:			
NREM	7.5 (4–28)	1 (0–11)	0.05 *
REM	21 (11–61)	2.5 (0–13)	0.05 *
TST	12 (6–36)	1 (0–13)	<0.05 *

* Statistically significant.
